# Constructing causal loop diagrams from large interview data sets

**DOI:** 10.1002/sdr.1745

**Published:** 2023-09-12

**Authors:** Pablo Newberry, Neil Carhart

**Affiliations:** University of Bristol, Bristol, UK

## Abstract

“Tackling the Root Causes Upstream of Unhealth Urban Development” is a trans-disciplinary research project seeking to map and understand urban development decision-making, visualise stakeholder mental models and codevelop improvement interventions. The project’s primary data was gathered through 123 semistructured interviews. This article applies, compares, and discusses four variations on a method for constructing causal loop diagrams to illuminate mental models and collective decision-making, based on manual and semiautomated processes applied to individual interview transcripts and datasets collected by thematic analysis. It concludes that while semiautomated approaches offer some time saving over manual approaches when applied to large data sets, care is required in interpreting and including peripheral contextual variables at the boundaries of the thematic analysis. Decisions regarding automation depend on the purpose of the modelling. Finally, the article recommends future applications record quantitative descriptors characterising the process of constructing CLDs from large qualitative data sets.

## Introduction

“Tackling the Root Causes Upstream of Unhealth Urban Development” (TRUUD) is a large-scale, mission-oriented, trans-disciplinary research project seeking to address the influence of the built environment on non-communicable diseases (NCDs) such as diabetes and poor mental health. Its overall aim is to codesign and test interventions that change the way decisions are made about urban development in order to reduce the incidence of noncommunicable diseases. One of the project’s initial objectives is to map and understand the systems of urban development decision-making, to visualise otherwise implicit mental models held by the key actors, and to codevelop interventions which improve the consideration of NCD risk factors and ultimately lead to better health outcomes. TRUUD brings together researchers from a variety of academic disciplines spanning urban development, transport, public health, real estate, management, public policy, law, and public involvement (Bates *et al*., 2023; Le Gouais *et al*., 2023).

The project set out to use causal loop diagrams (CLDs) to understand the underlying system of urban development decision-making and the various factors which influence the degree to which health is considered. The TRUUD project is motivated by findings from a precursor project that concluded upstream factors such as attitudes towards land use and land valuation, real estate financing, planning permission, and development delivery were critical in shaping urban and planetary health (Black *et al*., 2018). This research drew on urban-development stakeholders’ experiences to highlight descriptive reference modes observed from past behaviours. These include the ability of financial tools to appropriately value health, the duration of financial horizons, and the degree of coproduction partnership between the public, private and community sectors all persisting at a low level and playing a causal role in the similarly persistent low prioritisation of health within urban-development decision-making (Black *et al*., 2021). These findings shaped the design of the interviews. The process described in this article concerns a step in the production of preliminary CLDs to better understand and articulate the causal mechanisms behind these problematic behaviours (i.e., to develop a set of dynamic hypotheses as to their interaction and why they are persistently low that could be further developed and ultimately used to inform the design of potential interventions).

Although ultimately within the scope of the project, this article is not attempting to provide detailed analysis and commentary on the resultant models and the specific feedback loops they contain. Instead, it focuses specifically on challenges within the model-building process related to the construction of causal loop diagrams from large volumes of interview transcripts. These models form the start of further participatory modelling, refinement, validation, and verification. In some ways, the process described here aligns more with the mode of operation aimed at system conceptualisation (Lane, 2008), as set out by those such as Wolstenholme and Coyle (1983), appreciating that there are limitations to this qualitative mode of mapping to understand a system and advantages to preparing models for simulation that analyse a problem (e.g. Homer and Oliva, 2001; Lane, 2008; Wolstenholme, 1999).

As with many projects confronting complex real-world wicked problems, the project’s initial phase involved extensive engagement with experts, key decision-makers, and other public, private, and third-sector stakeholders. This consisted of 123 semistructured interviews across the problem space and four participatory workshops. While most interviews (112) were conducted with a single interviewee, some were conducted as small-group interviews. Interviews were carried out by 13 researchers operating in seven thematic teams, each interviewing stakeholders from a particular area as summarised in Table 1. The semistructured interviews (average length: 53 minutes; 43 seconds) generated approximately 900,000 words of qualitative data which were initially analysed through a process of thematic analysis. In summary, the researchers that conducted the interviews labelled segments of the transcripts with themes, then sorted these themes into 22 categories such as “Actor networks,” “Governance,” and “Public involvement,” which formed coding trees with subcategories nested within them. This article considers the method variations employed to construct causal loop diagrams from this data, representing individual mental models and mapping collective decision-making practices.

The article provides a brief overview of current practice for building such models from existing sources of text before applying, comparing, and discussing four variations on a method for constructing CLDs from the TRUUD data:

1. Manual data selection and manual construction of CLDs from individual uncoded interview transcripts.
2. Manual data selection and manual construction of CLDs from collected coded interview transcripts.
3. Semiautomated data selection and manual construction of CLDs from individual uncoded interview transcripts.
4. Semiautomated data selection and manual construction of CLDs from collected coded interview transcripts.

These are described as “variations on a method” because each variation follows the same six steps but varies in the way the steps are carried out and in terms of the start and end points of the process (as shown in Table 5). For Variations 3 and 4, the selection of relevant data segments from which to assemble the model is semiautomated because potential causal relationships are automatically identified based on user inputs, which then need to be validated manually by the model builder who must also still manually transform the text into microstructures and compile them into CLDs. The article considers the verification of the maps against the source text and the ease with which the resulting outputs can be validated through additional review (such as group model-building workshops), but the specific methods and processes of validation are out of scope. Overall, it seeks to observe and reflect on the experiences, challenges, and opportunities presented by these methods.

### The use of causal loop diagrams in public health

The system of interest for the TRUUD project concerns the intersection of urban development and public health. Systems thinking and modelling have long been advocated to complement other approaches and tools within public health (Leischow and Milstein, 2006). While many different systems methods have been used (McGill *et al*., 2021), causal loop diagrams and system dynamics models in particular have received increasing attention in understanding complex public health policy issues and designing associated interventions (Darabi and Hosseinichimeh, 2020). Many methods have been employed to construct the models including group model building (Andersen *et al*., 1997; Richardson *et al*., 1992), semistructured interviews, quantitative data analysis, and the use of preexisting documentary sources.

The focus here is on the creation of CLDs via the analysis of primary data collected by semistructured interviews. The resultant CLDs could be taken to group model-building workshops for refinement and validation, but this stage is out of scope for this article. Nevertheless, it should be noted that the benefits of GMB are well established.

Baugh Littlejohns *et al*.’*s* (2021) scoping review identified 23 peer-reviewed articles published between Jan 2018 and March 2021 focused on the use of CLDs in public health. Ten of these used interview data in the creation of the CLDs. They summarise the intended purpose of the CLDs in the 23 papers as:

- To illustrate complexity and identify leverage points
- To inform policy and practice
- To develop system dynamics models
- To measure and evaluate system change
- To enhance stakeholder and community participation
- To inform future research and enhance theoretical perspectives

The scale and focus of the modelling activities vary considerably. For example, Owen *et al*. (2018) conducted 16 interviews and analysed the transcripts using a process of purposive text analysis proposed by Kim and Andersen (2012) to generate CLDs with the aim of preventing obesity in children under 5 years old. Another study (Jalali *et al*., 2019) looking at public health interventions to tackle obesity, conducted seven semistructured interviews, producing 335 minutes of audio. The interviews were analysed through inductive and generative coding. Variables and their relationships were identified from this analysis (interview codes grouped to become model variables), and the models were validated with the interviewees and developed for simulation. At a much larger scale, Clarke *et al*.’*s* (2021) study into six obesity-prevention policy interventions utilised 57 semistructured interviews (16–75 minutes) into the policymaking process, together with the analysis of 568 additional documents and field notes. The text was examined through various forms of thematic analysis before following Kim and Andersen’s (2012) process. A CLD was generated for each intervention and then combined to a meta-CLD.

Baugh Littlejohns *et al*. (2018) investigated key factors influencing health promotion policy and practice in an Australian health system. They conducted 53 interviews, coded and analysed the transcripts and supporting documents, and summarised the key findings. Based on this, they adapted Kim and Andersen’s (2012) method to identify dominant themes present in both data sets and build a CLD, focusing on feedback loops that either facilitated or inhibited health promotion policy and practice. Bensberg *et al*. (2021) conducted 31 semistructured interviews (ranging from 23 to 120 minutes). They used thematic analysis to identify discussions in the transcripts and again the method developed from Kim and Andersen (2012) to identify the explicit or implicit relationships between them.

CLD-related approaches are also being utilised at the specific intersection of public health and urban decision-making. For example, Eker *et al*. (2018) investigate the interaction between housing, energy, and wellbeing via 17 semistructured interviews. A CLD was produced based on the method outlined by Eker and Zimmermann (2016) and then developed through three group model-building (GMB) workshops. Pineo *et al*. (2019) interviewed 22 participants to look at urban health indicators (UHIs) and their role in promoting health in urban planning policy and decision-making. They produced five thematic CLDs representing the participants mental models and validated these through a workshop with six participants.

Table 2 summarises the characteristics of the comparable studies mentioned above. The number of variables and causal links are also summarised as these may provide useful comparisons for the resultant models based on TRUUD’s primary data.

Several studies in public health have constructed CLDs using other approaches. Some, such as Burrell *et al*. (2021), used secondary data sources of first-person accounts combined with GMB. Others conducted interviews but did not use the direct transcripts (Parmar *et al*., 2021). Garvin *et al*. (2020) extract 1054 data segments from 47 research articles, giving rise to 2880 variables with 1440 causal connections.

With 123 interviews averaging just under an hour, TRUUD is faced with a significantly larger data set than the comparable examples in Table 2. It should be noted for context that while the intention to create CLDs from the interview data was known before the interviews took place, they were not designed with the sole intention of informing CLD creation. This was a significant factor in the volume of data collected. Other researchers have reported an average of 3 days to analyse and develop a CLD from 15 to 20,000-word transcripts (Valcourt *et al*., 2020). This would equate to around 45 days to analyse the 916,000 words from the TRUUD interview data. It is therefore of interest to reflect upon different approaches for the creation of CLDs from such data and consider their efficiency and efficacy. Before this, we dissect and compare methods of constructing CLDs from transcripts that are used in relevant studies. These approaches underpin the four variations on a method developed and applied in this research.

### Extracting causal loop diagrams from text

Various approaches for constructing CLDs from text have been proposed (Eker and Zimmermann, 2016; Kim and Andersen, 2012; Turner *et al*., 2013a) with some applied specifically in a public health context (e.g. Cassidy *et al*., 2022) as discussed in the previous section. This study is concerned with trialling and developing manual and semiautomated variations on a method with the aim of deciding how best to proceed in analysing a large qualitative data set.

This section outlines four related methods for inductively developing CLDs manually from interview transcripts as established and implemented by: (i) Kim and Andersen (2012), (ii) Turner *et al*. (2013b), (iii) Eker and Zimmermann (2016), and (iv) Baugh Littlejohns *et al*. (2018). The first two methods are selected as the most well established and highly cited methods in both the general system dynamics and CLD literature, as well as a majority of the public-health-focused applications discussed above. The second two methods were selected as the most prominent methods emerging specifically from the development of CLDs from interviews in the public health and urban development sectors. It also briefly summarises some key semiautomated methods for supporting these processes.

#### Manual methods

Kim and Andersen (2012, pp. 311-328) formalised a coding approach for generating CLDs from text, based upon grounded theory methodologies (see Glaser and Strauss, 1967; Strauss and Corbin, 1990, 1994). They aimed to build confidence in the CLDs by creating a traceable link back to the source data. They suggested the process is useful even if the source text was not collected by the modeller “nor intended to be used for the system dynamics modelling purpose.” Their process, often referred to as purposive text analysis, is summarised below:

1. Open coding of raw text data, categorised to discover themes and select relevant “data segments”
2. Identify microstructures of variables and their causal relationships within “data segments” in “coding charts”
3. Transform variables and causal relationships into “words-and-arrow” diagrams
4. Generalise structural “words-and-arrow” representations into a final causal map
5. Produce a data source reference table to link maps to the original text

Step 1’s “data segments” are text extracts (e.g. sentences or paragraphs) containing causal information. The “coding chart” referred to in Step 2 is a precursor to the visual representation of the CLD, taking the form of a table listing a cause variable, effect variable, and the nature of the relationship between them. Table 3 shows an example of a coding chart using TRUUD data. Generating coding charts can be labour intensive and time-consuming, with Kim and Andersen (2012) emphasising the need for a cost–benefit trade-off. This has led others to look at alternative approaches including elements of automation. Some look at providing additional guidance and procedures for this step, and others look to reduce this systematic formality.

Turner *et al*. (2013a) put forward an agenda for identifying the best ways to code interview data for the purpose of extracting CLDs based on their experience coding interviews, comparing the process established in Turner *et al*. (2013b) to Kim and Andersen’s (2012) group discussions. Table 4 compares these two highly influential methods with Eker and Zimmerman’s (2016) method, targeted at a systems audience, which aims to build on the strengths and weaknesses of the former two, and Baugh Littlejohns *et al*.’*s* (2018) widely cited method, targeted at a health-systems audience, which adapts Kim and Andersen’s (2012) method. The table outlines the key steps in the process of causal mapping from text data and highlights how approaches to undertake these steps differ between methods. In Step 1, “identifying concepts and discovering themes in the data,” all the methods take the raw text data (i.e. the input) and label it with themes (i.e. the output) through a process of open coding. However, Kim and Andersen use group-level data (i.e. transcripts from recorded group discussions), whereas Turner *et al*. and Eker and Zimmerman use individual-level data (i.e. transcripts from individual interviews). On the other hand, Baugh Littlejohns *et al*. use individual- and group-level data (i.e. transcripts from individual and group interviews). Furthermore, in Kim and Andersen’s method, the group members enter the meeting with a high degree of shared context and develop the context through discussion. In the case of the other three methods, the researchers leading the interviews set the context, so data collection may be highly targeted for the research purpose and grants the researchers greater control (Turner *et al*., 2013a). Open coding occurs for each method at this step, in which data is labelled with themes. Kim and Andersen, Turner *et al*., and Eker and Zimmerman follow a similar approach where codes identify various phenomena in the data, then are grouped with similar codes and named within categories. This is an iterative process that continues until dominant themes emerge. Baugh Littlejohns *et al*., on the other hand, code two data sets (i.e. policy documents and stakeholder interviews) according to their reference to elements in a unique coding schema and produce a summary of key findings from this analysis, which is the basis for the following steps.

At Step 2, “sorting or categorising themes in the data,” the methods begin with the same input — data labelled with themes — but apply different processes of axial coding that lead to diverging outputs; hence the step is divided into subrows. Kim and Andersen, Turner *et al*., and Eker and Zimmerman sort the data by themes into stakeholder groups, although it is implied by Kim and Andersen that this occurs as a second phase of open coding in Step 1. Beyond this, Eker and Zimmerman identify child nodes that express the content of data segments and aggregate these into themes, forming a hierarchical coding tree. Baugh Littlejohns *et al*. label key findings that are found in both data sets (i.e. policy documents and stakeholder interviews) as dominant themes to take forward to Step 3.

At Step 3, “identifying variables and their causal relationships,” both Kim and Andersen and Turner *et al*. identify causal structures within data segments and present them as coding charts, acting as a data source reference table. Kim and Andersen’s coding charts are from all the data segments compiled together whereas Turner *et al*.’*s* are compiled by themes within each stakeholder group, which allows for causal maps to be generated for each stakeholder group at Step 5 (pp. 311-328). Alternatively, Eker and Zimmerman identify causal relationships between child nodes (these act as the variables in this method) under a theme on NVivo, maintaining its reference to the data by linking it to the data source, which is less time-consuming than producing a reference table. The output is defined as a coding dictionary. Baugh Littlejohns *et al*. identify causal links between dominant themes and key findings, utilising Davidson’s (2000) proposed method: “including temporal precedence (i.e. establishing A before B), constant conjunction (i.e. when A, always B), and contiguity of influence (i.e. plausible mechanisms for linking A and B)”. Like Eker and Zimmerman, Baugh Littlejohns *et al*. identify variables and their causal relationships at a higher level than Kim and Andersen and Turner *et al*. who extract them from within the text.

At Step 4, “transforming text into words-and-arrow diagrams,” Kim and Andersen form words-and-arrow diagrams from the coding charts, whilst Baugh Littlejohns *et al*. form them from the causal interactions identified. Turner *et al*. and Eker and Zimmerman skip this step to generate causal maps at Step 5 directly from their coding charts and coding dictionary, respectively. At Step 5, “generalising structural representations,” causal maps are generated. Kim and Andersen generalise the words-and-arrows diagrams so that the system structures from different data segments can be merged into a causal map. Turner *et al*. create a rough draft of the causal maps for each stakeholder group from the coding charts by linking together repeated variables or like-variables. Once the causal map for each stakeholder group is complete, they are synthesised to create a single causal map. Eker and Zimmerman generate causal maps for each abstract theme using the list of relationships between variables in the coding dictionary, and like Turner *et al*., they are synthesised to create a single causal map. Baugh Littlejohns *et al*. form feedback loops from the words-and-arrow diagrams and assemble them into a single causal map.

At Step 6, “linking maps to the data source,” Kim and Andersen aim to build confidence in the causal map by creating a data-source reference table that matches conversation identification numbers assigned to each data segment and its coding chart with map identification numbers assigned to each causal link or variable in the causal map. As discussed in Turner *et al*. (2013a), the need for a data-source reference table is not essential for Turner *et al*. because the interviewer/coder’s direct contact with the data source, and therefore understanding of the interviewee’s intent and meaning, helps to reduce bias in the coding process. Baugh Littlejohns *et al*. also do not carry out this step. On the other hand, Eker and Zimmerman maintain a link between the map and the data source through their actions at Step 3 where relationships are recorded in NVivo and linked to the data source.

#### Semiautomated methods

Manually constructing CLDs from interview transcripts can be time consuming and resource intensive (Valcourt *et al*., 2020), leading many to seek methods to automate elements of the process. Yearworth and White (2013) adopt a constructionist interpretation of grounded theory in their method for developing CLDs from computer aided qualitive data analysis software (CAQDAS). Specifically, this utilises the matrix query function in the NVivo software package to identify potential causal relationships. The matrix query can be used to indicate the frequency with which any two coded elements are in a certain proximity of each other (for example, where they occur within the same paragraph). The resulting adjacency matrix may help the identification of data segments and relationships between codes in a large set. They offer a process for refining the matrix, though it remains the task of the researcher to interpret the relationships presented by the matrix query.

Others have explored full automation, natural language processing (NLP), and machine learning to seek causal statements/cause-effect pairs (Kontos *et al*., 2002; Pechsiri *et al*., 2019). These methods use linguistic, semantic, and syntactical analysis for causality, Bayesian inference, and methods developed to identify temporal relations (Nazaruka, 2019). Linguistic-pattern recognition-based approaches have been shown to be more easily interpretable than statistical methods and have been recommended for casual users and those seeking interactive enquiry (Hogenboom *et al*., 2011). Statistical methods and fully automated NLP-based approaches are beyond the scope of this article, although the semiautomated CAQDAS-based variations on the method discussed in the following section draw on some of the same seminal linguistic research on causal constructs (e.g. Altenberg, 1984).

### Application of method variations to construct CLDs from text

**Fig. 1:**
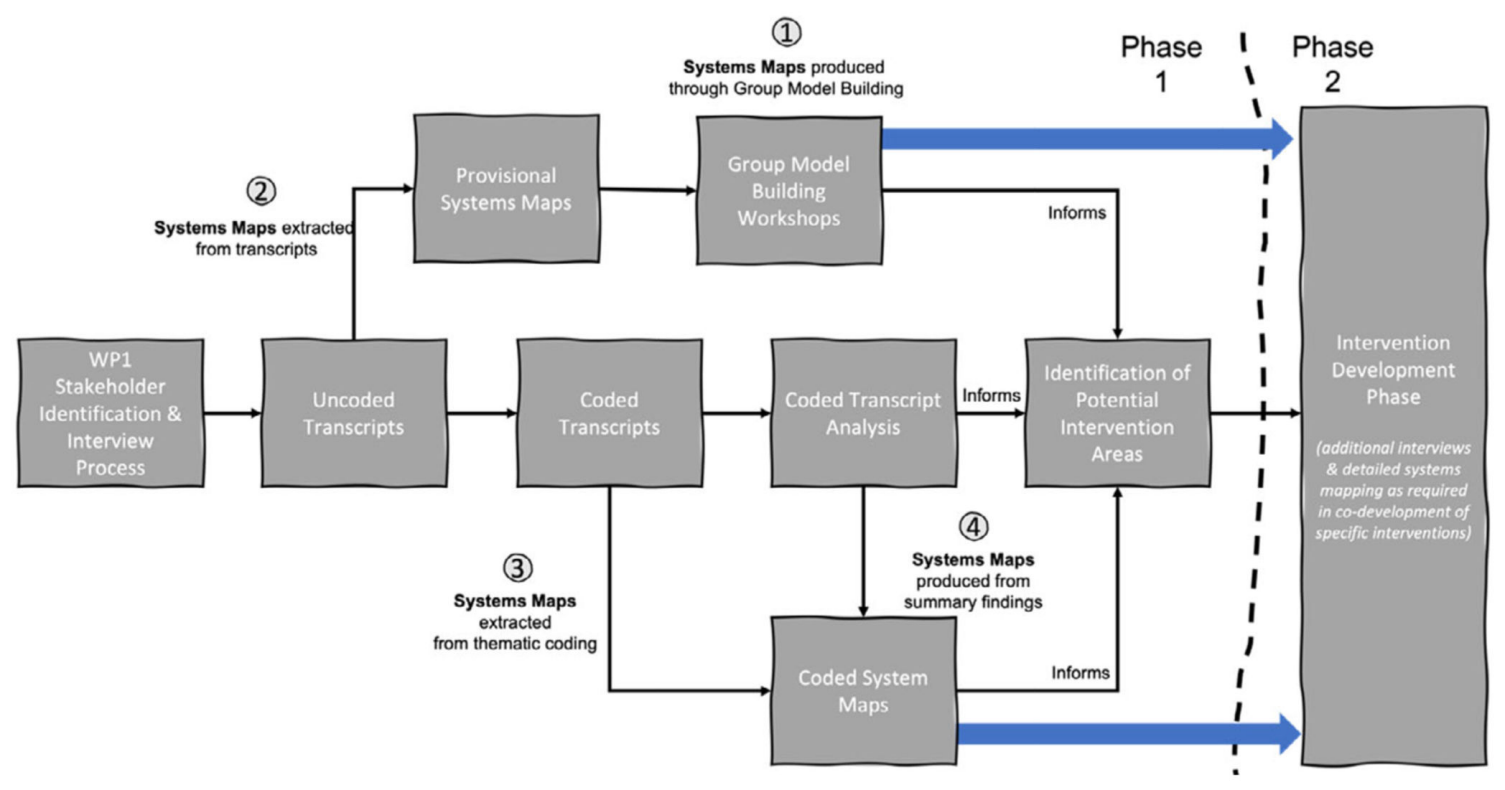
Outline of overall system mapping plan for TRUUD project

To gather information about the system of interest concerning health in urban decision-making, and specifically to explore the systemic reasons for the persistent low levels of factors which affect the consideration of health (such as the inability of financial tools used in urban development to appropriately value health and the low levels of meaningful collaboration between stakeholders), the TRUUD project facilitated group model-building (GMB) workshops and conducted extensive stakeholder interviews. The project undertook four routes to construct CLDs. CLDs were generated in GMB workshops (Route 1 in Figure 1). These were informed by provisional CLDs, each constructed from a single interview transcript that had not undergone collective thematic analysis (Route 2). This was necessitated by the workshops occurring in parallel with the interview process.

The method of manually constructing CLDs from each interview transcript was considered for the whole dataset of 123 interviews. This could offer value through the discreet representation of individual understandings, perspectives, and mental models, which could then be combined to collected or shared models. The value of this was considered against the cost of manually processing 123 transcripts with an average length of 7330 words and ultimately rejected on grounds of resource availability.

Once the interviews and thematic analysis had been conducted (Le Gouais *et al*., 2023), a method for manually constructing CLDs from each data set (the collected coded transcript extracts) relating to the 22 identified categories (or subcategories within them) was considered (Route 3). This option also requires considerable time and resources, hence the motivation to consider automation or semiautomation of the process. A fourth proposed route constructs CLDs from the analysis of the transcripts rather than the transcripts themselves.

This article concerns Routes 2 and 3, the construction of CLDs from the *uncoded* and *coded* interview transcripts. The CLDs were constructed by applying variations on a method using both *manual* and *semiautomated* processes. They are variations in the sense that each follows a general process in which there are alternative ways of undertaking each step. Some steps may be skipped if necessary/possible.

In this section, we describe the four variations. They can be contextualised through comparison to the methods previously discussed and shown in Table 4 (Baugh Littlejohns *et al*., 2018; Eker and Zimmermann, 2016; Kim and Andersen, 2012; Owen *et al*., 2018; Turner, Kim, and Andersen, 2013a). As such, Table 5 provides a comparison of the four processes using a similar framework to Table 4. To illustrate how Table 5 works, “Variation 1: Manual Data Selection and Manual Construction from Individual Uncoded Transcripts” starts at Step 3 of the process, simultaneously with Step 4, in which variables and causal relationships are identified to form microstructure maps. It finishes at Step 5 where the microstructure maps are merged to create a causal map. Step 6 is optional if variables and causal links are annotated in NVivo to provide a reference from the map to the data source. On the other hand, “Variation 4: Semiautomated Data Selection and Manual Construction from Collected Coded Transcripts,” includes every step of the process from using open coding to discover themes in the data at Step 1 through axial coding to sort themes in the data at Step 2 to finally linking the thematic maps generated at Step 5 to the data source using annotations in NVivo at Step 6. Each variation is described in detail in the following subsections.

Like Eker and Zimmermann (2016), Turner, Kim, and Andersen’s (2013) framework of six research design dimensions is used to explain how the variations compare to other studies. However, two additional dimensions are introduced related to modeller characteristics. Table 6 lists these eight dimensions and compares our coding approach to those previously discussed (Baugh Littlejohns *et al*., 2018; Eker and Zimmermann, 2016; Kim and Andersen, 2012; Turner *et al*., 2013b).

Most TRUUD project interviews were conducted individually (112 of 123) and are therefore considered asynchronous. While some had two or three people present, they are not considered synchronous group conversations in the sense used by Kim and Andersen (2012) or Baugh Littlejohns *et al*. (2018). This retained the option to construct CLDs representing individual mental models based on single transcripts and then synthesise into shared mental models around multiple stakeholder groups (see Table 1) or to construct CLDs representing shared mental models based on themes coded in the data.

Apart from Kim and Andersen’s (2012) study, where the purpose of the group conversations was separate to the purpose of the research, the data-collection characteristics of our study were similar to others in that the context was set by the researcher and data collected by the researcher. Furthermore, the interviewees represent many subgroups within the system of urban-development decision-making.

Thirteen researchers undertook the interviews (six of the interview teams consisted of two researchers each and one team consisted of one researcher), 10 of which went on to code the transcript data. This research took steps to ensure the “convergence of multiple coders.” Turner *et al*. (2013a, p. 258) employed a coherent set of perspectives to code the purposive text data, achieved intercoder reliability, and integrated the coding results systematically. Within the study reported here, double coding was completed in pairs and matrix analysis undertaken within NVivo to cross-check coding style, indicating a similarity between coders of 90%.

This study diverges in relation to modeller characteristics. Here, the model builders were neither engaged in the data collection nor the coding. While the model builders had relevant expertise in the field of study, they were not familiar with the interview transcripts or coding before constructing the CLDs. It could be argued this risks a lack of sensitivity around nuances in the data but also that it provides the advantage of reducing bias in the interpretation of the data. Furthermore, having a separate dedicated modelling resource within the project enabled the other researchers to continue their analysis of the coded data, in parallel.

**Fig. 2:**
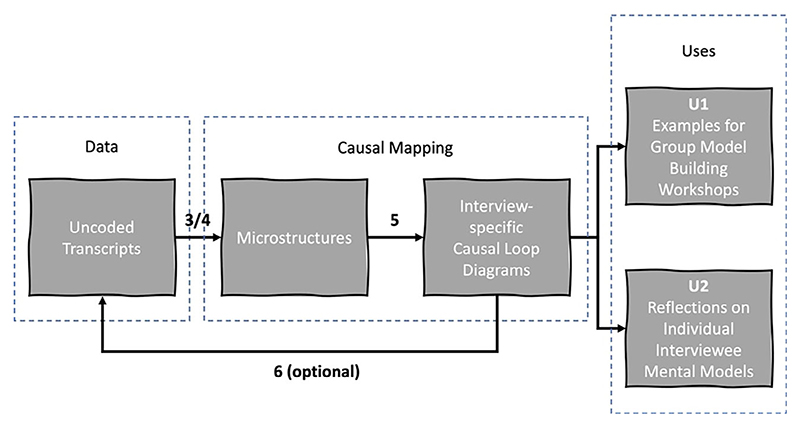
Variation 1: Manual data selection and manual construction of CLDs from uncoded transcripts

Deploying variations on a method enabled CLDs to be constructed and used for different purposes during and after the data coding process. At each stage, decisions regarding the type of variation applied considered time, resource, and need. Table 7 summarises the five ways CLDs were used (U1 to U5), as identified for each variation in the following section (Figures 2, 4, 7, and 9).

Quantitative descriptive characteristics regarding the resultant CLDs from each variation are also provided. This is intended to add to the growing set of data in this field and encourage others to report similar data. Experimenting with and evaluating these variations, as well as benchmarking the performance of individual coders and model builders, offers insights that could inform future research projects looking to construct CLDs from interview data sets. Moreover, the conclusions from the study reported here will inform the construction of CLDs as the TRUUD project continues. While example CLDs produced through the application of the method variations are shown in the following sections, they are recognised as incomplete outputs from the data at this stage. Therefore, their detailed analysis, including reflection on their function as dynamic hypotheses explaining the previously observed reference modes is out of scope, while reflections are made on the process of applying the method variations. Consideration is given to whether variables, causal connections, and feedback loops are being identified through the variations, but the meaning or value of the feedback processes are not discussed as they are based on incomplete data.

#### Manual construction of CLDs

Variation 1 involves the *manual* data selection and *manual* construction of CLDs from *uncoded* transcripts, while Variation 2 concerns the *manual* data selection and *manual* construction of CLDs from *coded* transcripts.

*Variation 1* — *Manual data selection and manual construction from individual uncoded transcripts*: The process is illustrated in Figure 2. At this stage, the transcripts had not yet been coded by researchers, therefore the variation begins at Step 3/4 as described in Table 5.

To construct CLDs using this approach, the model builder read the interview transcripts once to become familiar with the content, then directly (i.e. without an interim coding chart) mapped the variables and perceived causal links between them into Vensim as microstructure maps.

The model builder was vigilant for implicit as well as explicit causal relationships. Altenberg (1984) provides examples to demonstrate the difference between them: an explicit causal relationship might use an overt causal “link,” such as *because* — “John is in hospital, because he had an accident” — whereas an implicit causal relationship might use, for example, juxtaposition: “John had an accident. He is now in hospital.” Furthermore, Altenberg (1984, p. 21) concluded that “speakers seldom have time to present causal events in a logical or thematic order (p. 68).” As will be seen in the following section, the manual approach is more likely to identify implicit causality than the semiautomated approach used in this research. While the manual approach has the flexibility to capture the nuance and richness of the interview data, it comes at the cost of time, methodological rigour, and reliance on the interpretation of the model builder — the latter of which can lead to cognitive biases.

As applied, Variation 1 did not provide a traceable link between CLD elements and the original data source, but it would be possible to record such annotations directly into the NVivo file (as per Step 3 in Table 5). As each CLD is based directly on a single interview transcript, it can therefore be traced back to its source with relative ease. If CLDs from individual transcripts were to be combined into a CLD of more than one mental model, then each variable would need to be tagged with the transcript ID to maintain a level of traceability.

**Fig. 3:**
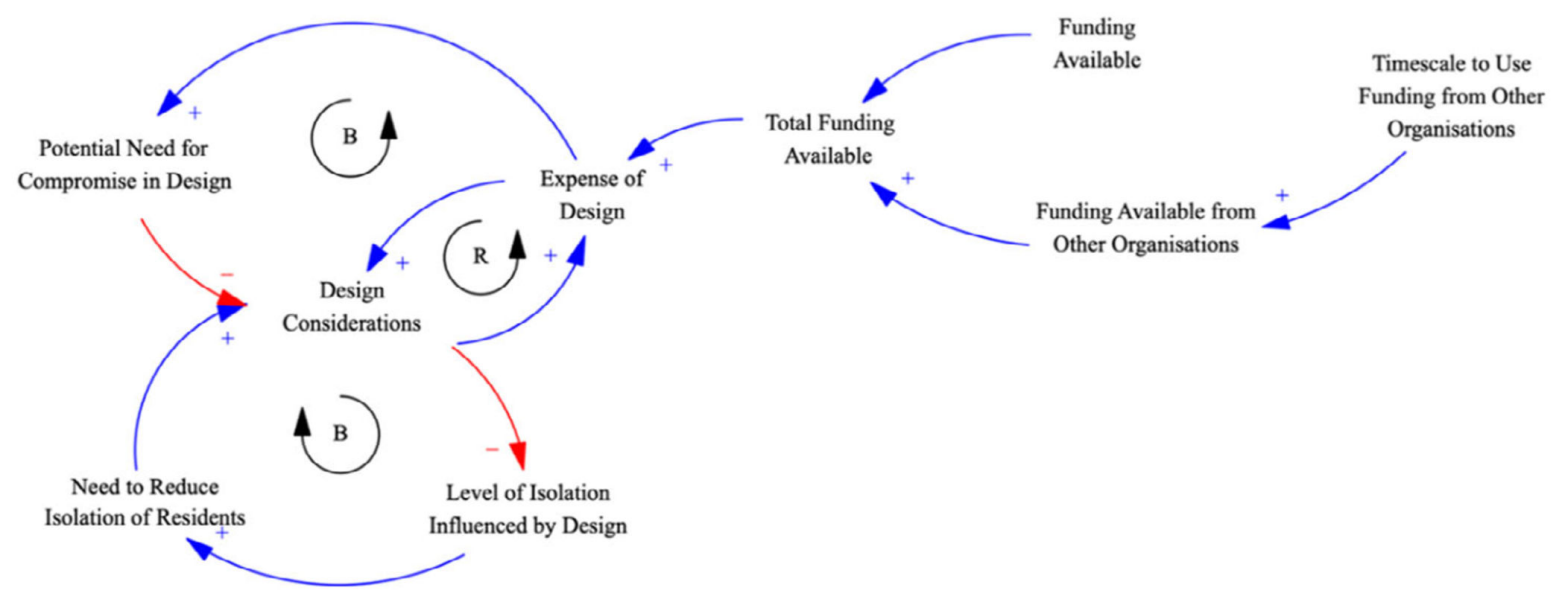
Larger microstructure with feedback loops constructed manually from uncoded transcript

To demonstrate Variation 1, it was applied to a transcript of 7066 words. From this, 123 variables were identified (0.0174 variables per word), and 27 microstructures were extracted (0.0038 microstructures per word). Fourteen of the microstructures included four or more variables, and six feedback loops were identified in total. Figure 3 illustrates one of the larger microstructures, including balancing and reinforcing feedback loops, that represent the following segment of the transcript (variables highlighted in bold brackets added):

“We were very careful about the design because we didn’t want people to be stuck in a little flat with no ability to talk to neighbours or see what’s going on outside **(Level of Isolation Influenced by Design)**, so our actual design ended up a lot more expensive than it should have been **(Expense of Design)** because we were taking a lot of these factors into account **(Design Considerations)** and we may have to do some compromises **(Potential Need for Compromise in Design)**, but at the beginning, that was our aim, to make sure that everybody didn’t feel trapped in a little box **(Need to Reduce Isolation of Residents)**. […] predominantly because we’re the ones that are funding most of this **(Funding Available)**, but I think we’ve got money coming from ((organisation name removed)) and some other places **(Funding Available from Other Organisations)** but we are on a tight timescale because some of these are time limited **(Timescale to Use Funding from Other Organisations)**.”

As can be seen by comparing the text to the CLD, causality has been assumed as implicit where no explicit causal linking word, such as *because* or *so*, has been used. Nevertheless, these connections seem logical in the context of what the interviewee was saying. These causal links are potentially contentious. However, because Variation 1 captures implicit as well as explicit causality, it has the potential to extract microstructures with higher detail complexity (i.e. more variables and links) and a more detailed reflection of the interviewee’s mental model. It can be argued that a subsequent review by the interviewee would validate this and, because of its greater complexity, provide more opportunity for further development of the causal map and understanding of the topic than a simpler model might.

Variation 1 generated CLDs to be used as examples for GMB workshops (U1), which acted as a separate parallel data-gathering step to the interviews. The purpose of the CLDs was to contextualise them in relation to aspects of the urban-development decision-making system and thus help stakeholders understand the logic of the models related to their area of expertise. Furthermore, the CLDs provided the basis of potential discussions and geared stake-holders to actively participate in the model-building exercises. The discussions based on the CLDs provided were not the primary focus of the workshops. Although it may have been methodologically advantageous to use the coded transcripts rather than the raw uncoded transcripts, these were not available with sufficient lead time to prepare for the workshops. Additionally, the CLDs simply provide a visualisation of each interview (U2). No observations or conclusions were drawn from the CLDs generated by Variation 1 prior to the GMB workshops. Such observations and conclusions pre- or post-GMB workshop, for example, in relation to explaining the observed reference modes are not within the scope of this article.

**Fig. 4:**
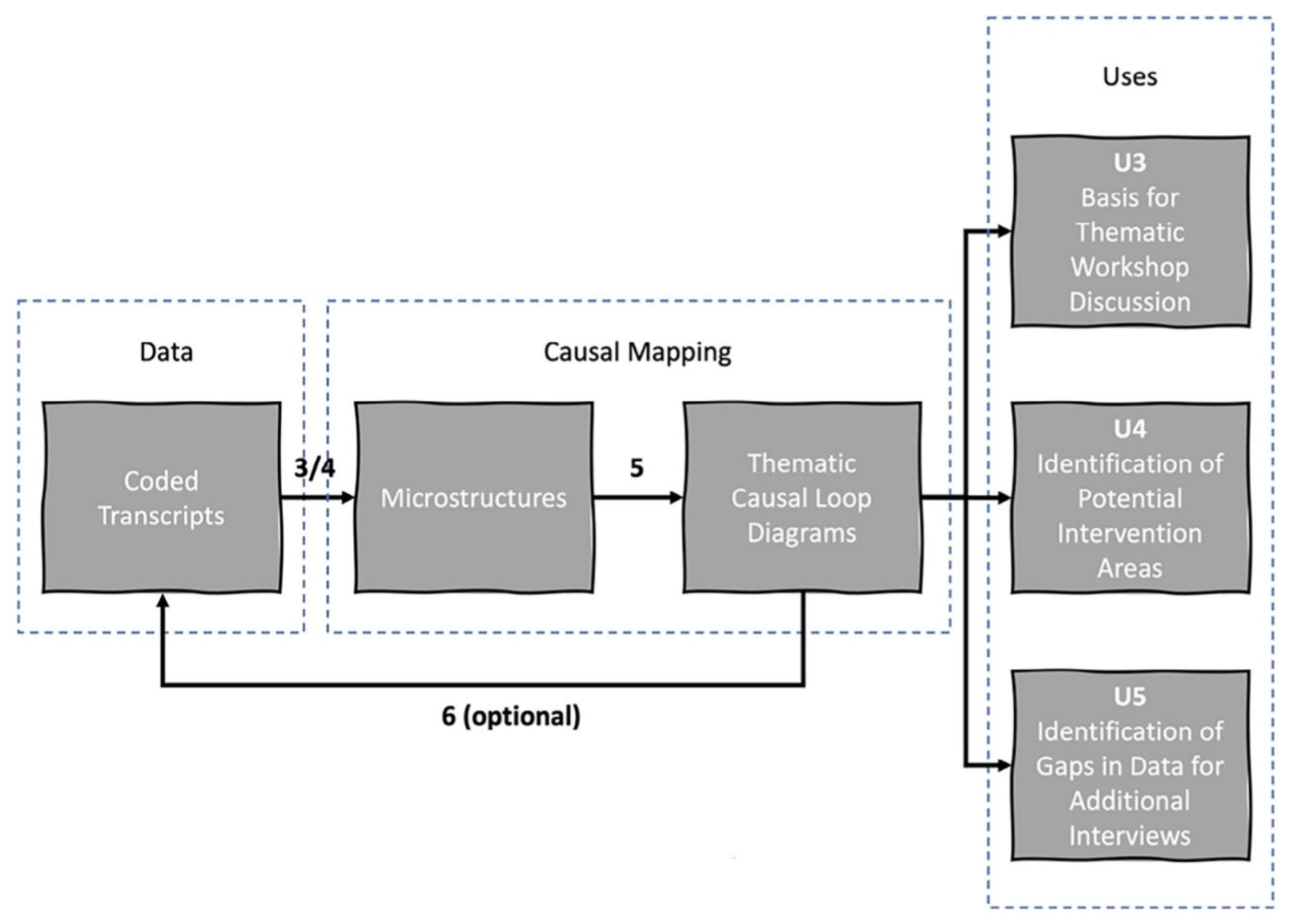
Variation 2: Manual construction of CLDs from coded transcripts

*Variation 2* — *Manual data selection and manual construction from coded transcripts*: the *manual* approach was also applied to a collection of thematically *coded* transcripts. The coding approach, described earlier, is outlined as steps 1 and 2 of Table 5. Figure 4 represents the process and Table 5 compares it to the other variations applied in this research.

The CLD produced in the example described here was used as the basis for a thematically focussed workshop discussion around the subcategory “coproduction — design — delivery” (U3 in Table 7/Figure 4). This sub-category, used during the open coding phase, was located under the “Public involvement” category/theme. There were 52 data segments across 20 transcripts tagged with this subcategory, totalling 8632 words. The first step of system mapping involved extracting microstructures from the data segments directly into Vensim. In the same way as described for Variation 1, implicit causality between variables was assumed in some instances where no explicit causal word was identified, as in the example data segment below (variables added in bold brackets):

“[…] you need a much more involved, deeper, deliberative forms of community engagement **(Deliberativeness of Community Engagement)**. All the best places **(Quality of Place)** are designed in cocreative processes **(Cocreativity of Design Process)** with local people actively involved.”

**Fig. 5:**
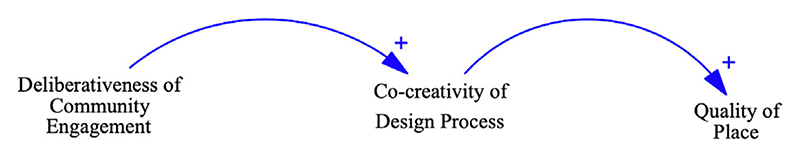
Microstructure constructed from implicit causal links in transcript segment

This suggests that with respect to urban development processes, deliberative community engagement is needed. Furthermore, the quality of a place can be influenced by a cocreative design process where local people are actively involved. It can be understood as implicit within this context that the deliberativeness of community engagement influences the cocreativity of the design process. Figure 5 represents the microstructure extracted from this segment.

**Fig. 6:**
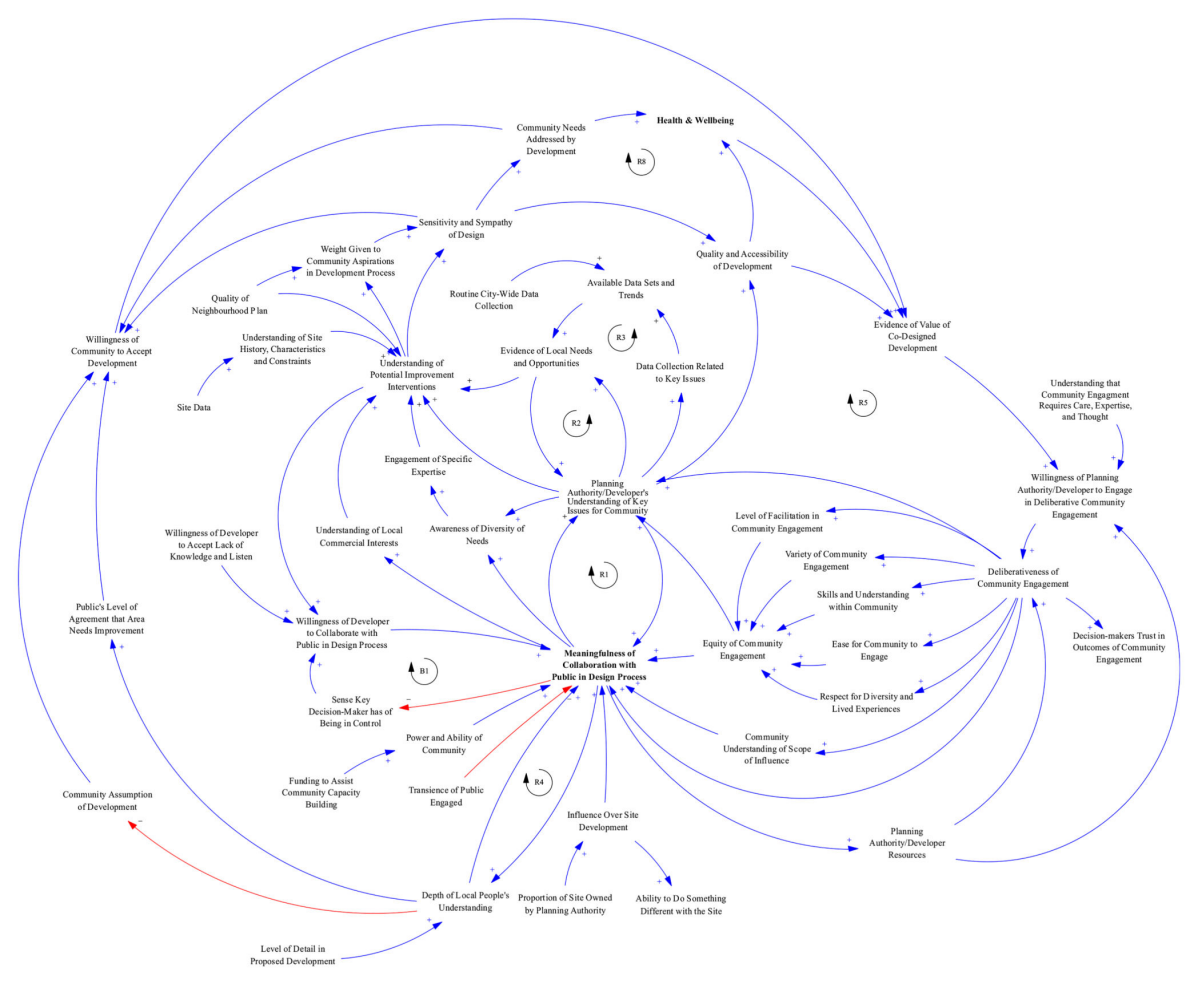
CLD manually constructed from the thematic code “Coproduction — design — delivery”

As a result of this process, 133 variables were identified (0.0154 variables per word), and 26 microstructures were extracted (0.003 microstructures per word). Only one feedback loop was identified in the microstructures. The microstructures were merged to construct a single CLD. This was an iterative process involving three iterations between a primary and secondary modeller. This collaboration aimed to reduce bias in the process of merging microstructures and interpreting causal relationships. Most microstructures had overlapping variables and/or perceived causal links between them because they were extracted from transcript segments with the same thematic code. Some variables were combined and expressed as a single variable that captured the meaning of the originals, and others were omitted if considered peripheral to the thematic focus on “coproduction — design — delivery” despite originally having been captured in text data segments under that code. The CLD was reviewed by two separate researchers with expertise in the involvement of the public in urban planning and health decisions, before being used as the basis of discussion on the theme of citizen engagement. The final CLD included 45 variables (Figure 6).

It is perhaps notable that in this application of Variation 2 many small, fragmentary microstructures, almost entirely made up of cause-effect pairs and short cause-effect chains, combine through their common variables to form a larger model with more detail complexity, including feedback loops. This may arise as an artefact of the coding process whereby many small data segments were produced, each leading to a small microstructure from a particular viewpoint. As the data segments are linked by theme, they have an increased likelihood of sharing common variables. The different perspectives from the interviewees are such that their partial viewpoints provide a partial view of a larger feedback process.

Only a subsection of this CLD was ultimately used in the workshop, although simplification through the removal of exogenous variables was considered (as used by Owen *et al*., 2018). Nevertheless, focusing on a thematic code integrates perspectives from a range of interviewees on a particular theme and presents their combined (but not necessarily shared) mental model.

#### Semiautomated data selection and manual construction of CLDs

Variation 3 was the *semiautomated* data selection and *manual* construction of CLDs from *uncoded* transcripts while Variation 4 was the *semiautomated* data selection and *manual* construction of CLDs from *coded* transcripts.

**Fig. 7:**
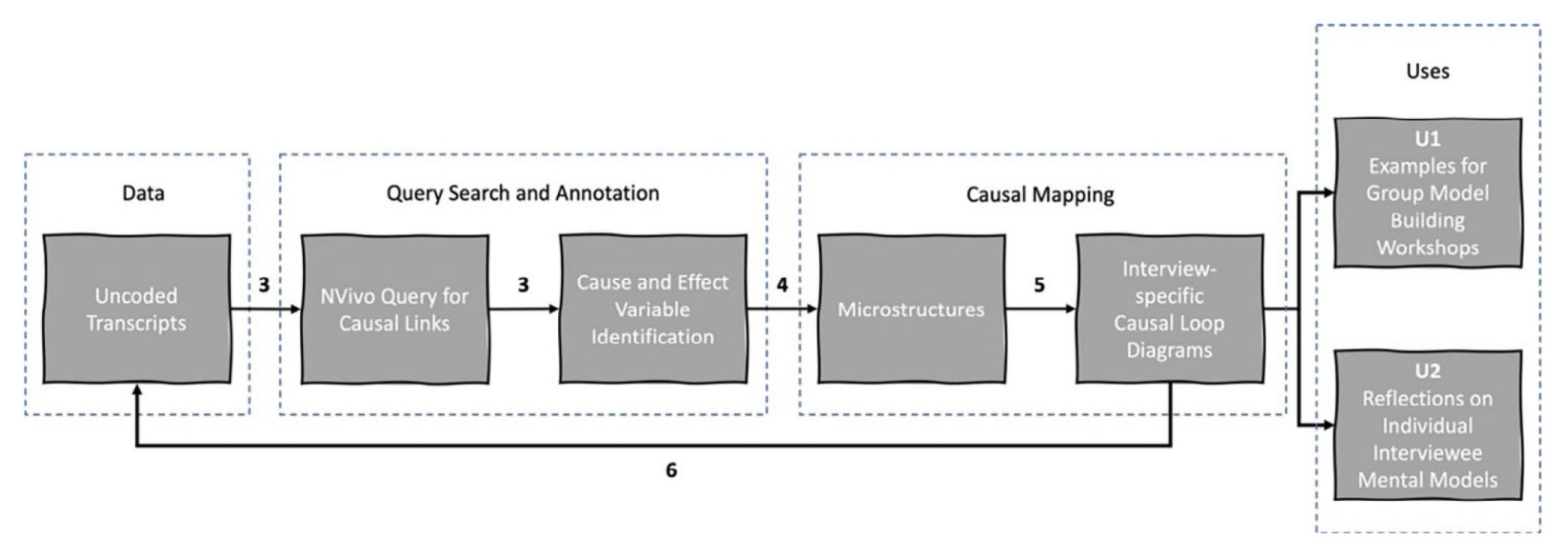
Variation 3: Semiautomated data selection and manual construction of CLDs from uncoded transcripts

*Variation 3* — *Semiautomated data selection and manual construction from individual uncoded transcripts*: This approach is shown in Figure 7 with the numbers referring to the steps outlined in Table 5. The semiautomated approach had a greater level of systematic formality and rigour than the manual approach.

Variation 3 utilised the “Text Search Query” function in NVivo to search for expressions of causal relations in uncoded transcripts. Firstly, literature on causal expressions was examined to generate a list for the text search criteria. Altenberg (1984) provides an inventory of causal expressions in spoken and written British English, including adverbial linkages (e.g. *so, hence, therefore*), prepositional linkages (e.g. *because of, on account of*), subordinations (e.g. *because, as, since*), and clause-integrated linkages (e.g. *that*’*s why, the result was*). He found that the expression of causal relations was more varied, lexically, and grammatically in the written corpus than the spoken corpus, which was more restricted with a high frequency of *so* and *because* (including its shortened form, *cos*) that together accounted for 74% of links (Altenberg, 1984). This indicated that *because, so*, and *cos* (typed as *cause* in interview transcripts) would be the most common causal expressions found by the search query.

The initial list of causal expressions was generated from Altenberg’s inventory with some simplification (e.g. Altenberg discusses different contexts, such as “*the result was*” or “*as a result*” which were collectively represented by the term “result” in the search). Furthermore, selecting the option to “include stemmed words” in the NVivo search query ensured that further expressions, such as *resulting*, would also be found.

**Fig. 8:**
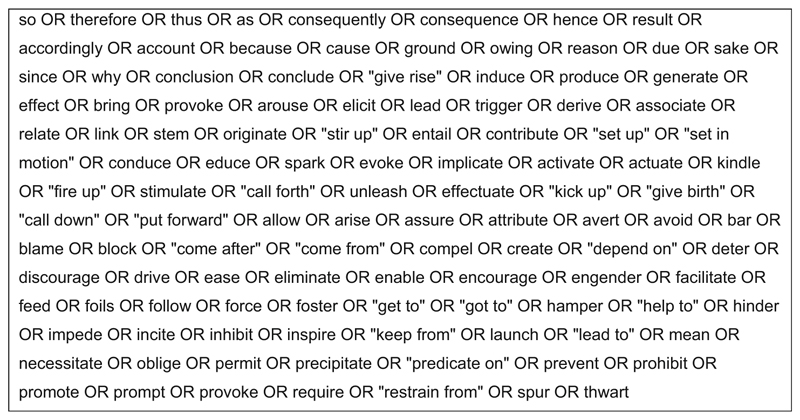
NVivo text search string for causal expressions (stemmed words included, e.g. “effecting”) based on Altenberg (1984), Girju and Moldovan (2002), and Dunietz (2018)

A second layer of causal expressions was added to the query from Girju and Moldovan (2002), who identify ambiguous causation verbs. These were all included in the search string. In this instance simplification was not possible as it would return a high frequency of noncausal expressions, so instead the specific expression sought was put in quotations to search for an exact match. One example is the inclusion of “*set up*” and “*set in motion*” in the search string but not *set*, which is not causal in and of itself. Furthermore, Dunietz *et al*. (2015) present an annotation scheme for causal language where *causal language* refers to “clauses or phrases in which one event, state, action, or entity (the cause) is explicitly presented as promoting or hindering another (the effect). The cause and effect must be deliberately related by an explicit causal connective.” Building on this, Dunietz (2018, p. 119) provides a comprehensive table of “connective words” to enable annotators to extract causal language from corpora. This was used to add the third and final layer of causal expressions to the list. Figure 8 shows the search string of causal expressions used in NVivo.

By running this text search query on a transcript in NVivo, all the words and phrases listed in the query were highlighted. The next step was to go through the transcript and identify the words or phrases being used as causal expressions and the cause-and-effect variables related to these. Where causal links were identified, the transcript was annotated directly in NVivo. Causal links with positive polarity were annotated with “> (+)” and causal links with negative polarity with “> (−)” with the arrow indicating the direction of causality. To demonstrate an example, one interviewee stated:

“They’re very commercially minded *so* whilst they might have aspirations in certain ESG categories, they’re there to make a return.”

The text search query highlighted the overt causal link, *so*, which the model builder used as a point of reference to identify potential variables either side. It was understood as explicit that an increase in “Commercial Mindedness of Organisation” led to an increase in “Desire to Make a Return.” Yet the model builder perceived an implicit causal relationship: that an increase in “Desire to Make a Return” may lead to a decrease in “Aspirations in ESG Categories.” Therefore, the segment was highlighted and assigned the following annotation in NVivo:

Commercial Mindedness of Organisation > (+) Desire to Make a Return > (–) Aspirations in ESG Categories.

Each microstructure identified was annotated in NVivo then mapped in Vensim. NVivo annotation creates a traceable link to the original data and increases confidence in the maps. Annotations of variables can be searched for within across transcripts.

As discussed in the previous section, this search query would not return any implicit causal links which are described by an interviewee without the use of any words explicitly associated with causality.

**Fig. 9:**
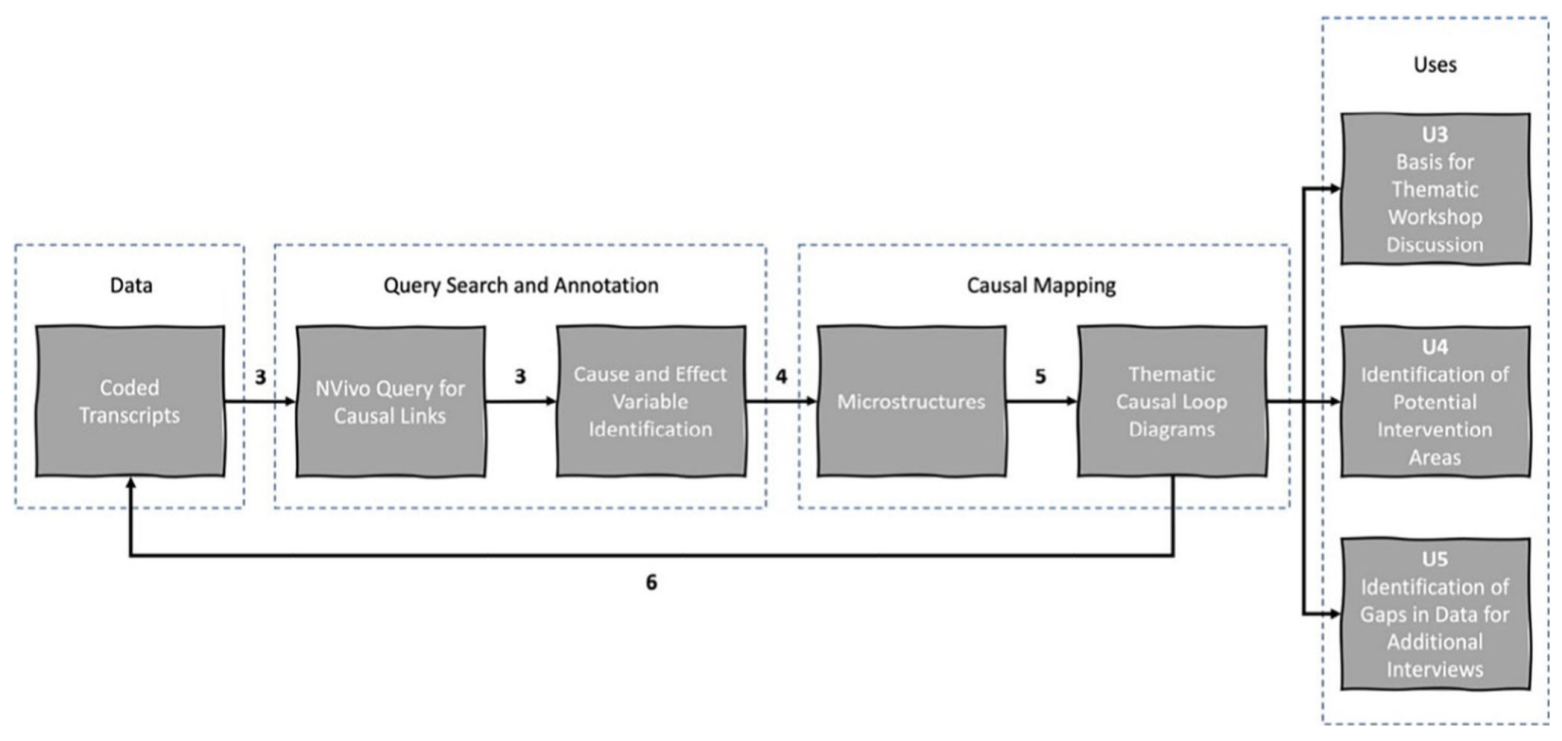
Variation 4: Semiautomated data selection and manual construction of CLDs from coded transcripts

*Variation 4* — *Semiautomated data selection and manual construction of CLDs from coded transcripts*. This is shown in Figure 9 and detailed in Table 5. Like Variation 3, it applies the text search query function in NVivo to search for expressions of causal relations. In this case, it was used to search for causal expressions within thematic codes — either at the category/ theme level or the subcategory level — rather than within uncoded transcripts. Therefore, it identified any causal expressions contained within data segments tagged with the selected code. Consequently, any coded data segments that did not contain a causal expression within the text search query, would not be presented to the model builder.

The text search query shown in Figure 8 was executed on the subcategory “Codesign — production — delivery.” This provided a comparison with manual Variation 2. The search query identified 284 potential causal linking words and phrases. By filtering out those that were not used causally (e.g. “so” used at the beginning of a sentence, not as a causal transition word) and, at this stage, ignoring whether the context was relevant to the aims of the causal mapping, 145 causal expressions were identified. The number of actual causal expressions identified equated to 51% of the words and phrases identified by the text search query (i.e. 49% were false positives). As the CLD for this subcategory had already been constructed using Variation 2, the cause-and-effect variables related to the causal expressions were not identified. Instead, to draw comparisons for the evaluative objectives of this exercise, it was assumed that there would be one cause and one effect variable per causal expression (i.e. two variables per causal expression). There is of course a possibility that the exact same variables could appear beside multiple causal expressions, but familiarity with the wide-ranging topics covered by the transcripts through the manual process described above supported the assumption that this was negligible at this stage (although some variables could be grouped and consolidated at later stages). As such, 145 was multiplied by two, resulting in an *estimated* 290 variables (0.0336 variables per word) in the subcategory.

In comparison, Variation 2’s manual approach identified 133 variables (0.0154 variables per word) in the subcategory, 46% fewer than estimated by applying Variation 4’s semiautomated approach.

On the surface, this contradicts the emerging findings that suggest the manual variations on the method (Variation 1 and 2) tend to extract more variables per word than the semiautomated variations (Variation 3 and 4), by virtue of their ability to identity implicit causality. However, this trial of Variation 4 did not account for the filtering stage, seen in Variation 2, whereby the model builder would not record any off-topic discussion. The disparity in the number of variables identified using Variation 2 and the estimated number of variables identified using Variation 4 can be explained largely by the number of false positives identified by the semiautomated data selection steps, but may also suggest there is a significant amount of off-topic discussion in the transcript data segments coded with any given category/subcategory, the modeller following the manual variation was less effective at finding causality, or the assumptions for the estimate are incorrect. This is considered in the final discussion of the paper.

#### Quantitative characteristics of CLDs constructed with manual and semiautomated approaches

This section provides the quantitative characteristics for six CLDs constructed from different coded and uncoded interview transcripts with manual and semiautomated approaches. The interview transcripts for each application of the method variations were selected for analysis at random from the overall data set of 123 interview transcripts. It was not possible to apply each Variation to the exact same data set (i.e. the same subset of interview transcripts) as it was felt the model builder would begin to introduce their prior knowledge of the data and the causal structures within it to later applications of Variations. As such, different data sets (i.e. different subsets of interview transcripts) were used as the input for each Variation. Using different data sets as inputs for each of the Variations presents challenges for direct quantitative comparison. While reporting the quantitative differences in both input data and the resultant outputs may help contextualise differences, the role in normalising for comparison is limited due to other qualitative variations that would be expected to exist between two different interview transcripts. Reporting this data is therefore done with the intention of being transparent about variation in the volume of data inputs and the characteristics of the outputs between the approaches. It is not a comparative assessment.

Table 8 records the quantitative characteristics of three fully manual examples (two using Variation 1 and one using Variation 2) and three semiautomated examples (all using Variation 3). As shown, the number of words in the text from which the CLDs were constructed ranges from 5680 to 10,552 words. The data is purely illustrative of the experiences on this project, and any observations are limited by an expected degree of natural variability between interview transcripts and thematically linked data segments.

To draw meaningful insights, a study could be designed to compare the application of manual and semiautomated approaches to the same interview data, although it should be recognised that the model builder may be influenced by their previous experience with the text. However, this article encourages others to report similar quantitative characteristics to add to a growing set of data in the field that can help to inform the design of future studies constructing CLDs from interview data.

## Discussion on CLD construction methods applied

Observations were made during the application of the four variations to the method which will be discussed in this section. The first set of observations relate to coded and uncoded transcripts and the way in which the coding associated with qualitative analysis is used in the construction of CLDs (both in these methods and the methods applied by others). The second set of observations relate to how and when in the process to generalise themes and variables. General issues of causal language in speech are also discussed along with other challenges in the construction of CLDs from interview transcripts. Finally, this section considers the overall efficacy of the variations in supporting the project to achieve its aim of better understanding the causal mechanisms behind the persistently low consideration of health in urban planning and the subsequent design of policy interventions.

### Coded vs. uncoded CLD extraction

**Fig. 10:**
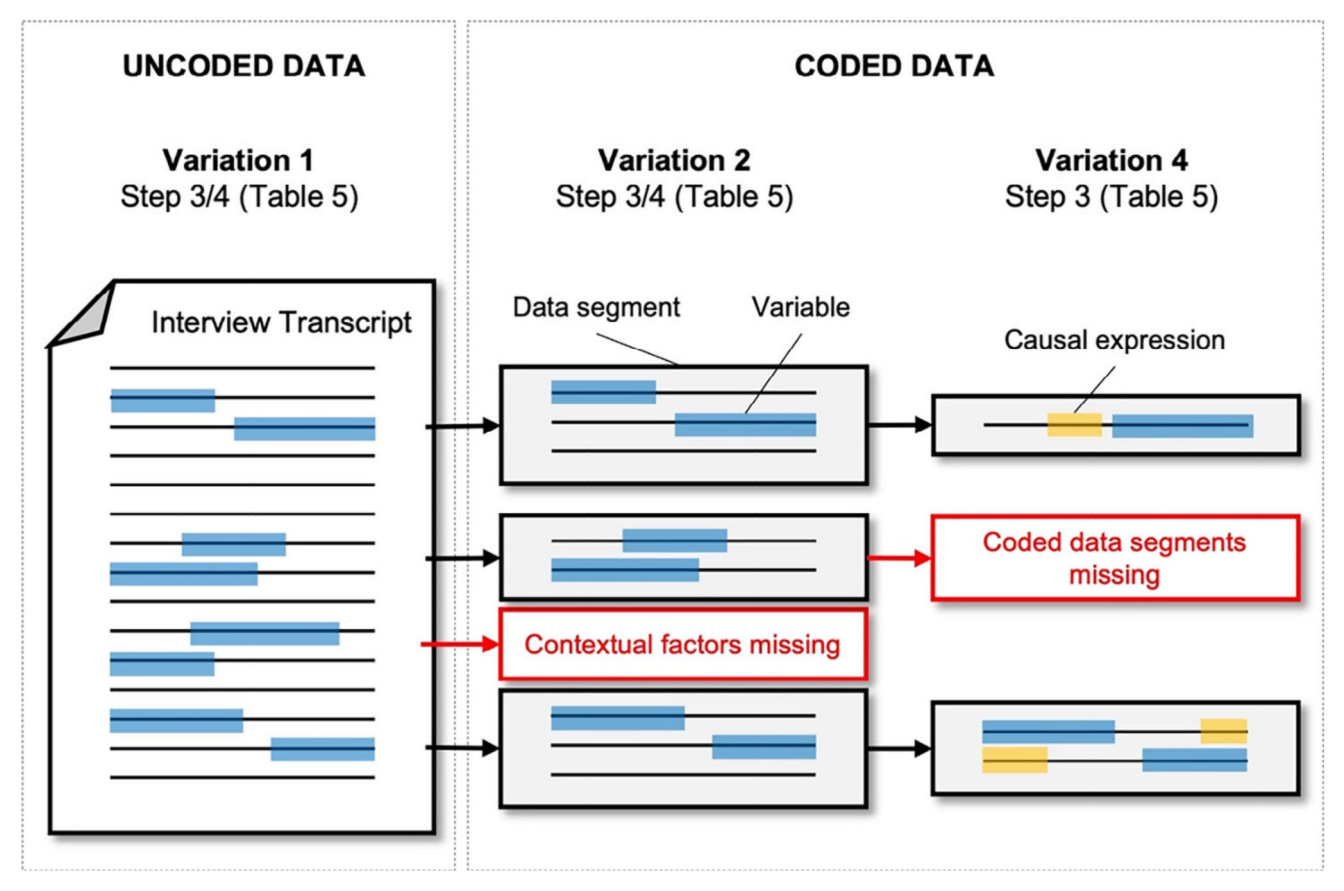
Diagram illustrating how Variations 2 and 4 filters information through coded data segments

The uncoded transcripts provide the wider context in which the interviewee is talking. A coded segment may only capture a fragment of what might be part of a longer chain of variables or even a complete loop if the whole transcript is analysed. The thematic coding can dictate a boundary for the system, but this may not be the most appropriate boundary for the construction of the model. Therefore, it is worth being aware that there might be factors outside those captured by the thematic code that are influencing the system, as is illustrated for Variation 2 in Figure 10. This may be more prevalent in situations where the thematic coding was not done by the modellers or with the purpose of modelling in mind. However, when extracting CLDs from a large data set in particular, boundaries do need to be set to handle complexity and to focus on specific topics of interest.

Furthermore, as the model builder was not involved in the data collection or coding in this research, at times it was difficult to understand the meaning of variables without referring to other parts of the transcript outside the coded data segment. For example, in one transcript the interviewee explains what they mean by “commercial opportunities” later in the transcript, and in another transcript, what was meant by “understanding” and “process” was not clear until reading around the coded data segment.

To overcome this challenge, the model builder could become more familiar with the detail of the interview transcripts. However, in this research, the data set is too large for model builders to investigate the context surrounding every coded data segment given project timescales. A pragmatic solution requires only the coded data segments not fully understood to be referred to their context in the original transcript.

A key consideration arises around what is lost as a result of choices to bound the body of text (such as using only segments with a specific thematic code or only segments with explicit causal language) and what is saved in terms of the time required to construct a model from the entire transcript? If the basis for the models is a relatively small set of transcripts, while thematic analysis remains useful, it may be more useful to construct the model from the complete body of text without extracting coded data segments around a given theme. Where the data set is larger it may be impractical to use the entire body of text. Similarly, representing a single mental model from an individual’s interview transcript could be done independently of any thematic analysis, although again this thematic analysis would remain useful for understanding the context of the model.

In summary, when utilising a large body of text to produce CLDs based on a collective or shared understanding of an emergent theme/category, then coding provides a useful means to condense the data set but presents issues with regards to bounding the system too early in the process and losing some contextual influences and understanding as a result. Furthermore, the semiautomated steps further reduce the size of the analysed data. This raises questions as to the value of collecting such large data sets in the first place, although these processes are still relevant if faced with preexisting data collected for other or multiple purposes as is the case here.

### Cognitive biases

Cognitive biases, such as salience bias and biases resulting from heuristics, are more likely to occur following the manual approaches (Variation 1 and 2) than the semiautomated approaches (Variation 3 and 4) because they involve a greater level of interpretation from the model builder. These cognitive biases can influence where implicit causality *is* and *is not* considered (whereas the semiautomated approaches exclude most incidences of implicit causality). Salience bias can draw attention to prominent or emotive details (i.e. salient information), whilst ignoring potentially important ones (Tiefenbeck *et al*., 2016). On the other hand, heuristics are mental shortcuts based on knowledge or expertise that can lead to quick probability judgements that risk systematic and predictable errors (Dale, 2015; Tversky and Kahneman, 1974). There are strategies that can be employed to address these cognitive biases in CLD construction. It is proposed as a first step that the model builder be aware of the bias at play, but awareness is not enough. They must also retain an open mindset, consider the full range and detail of the text, avoid making quick judgements, and, if possible, consult with other research team members in real time. Once causal links have been established, the model builder can seek feedback from other research team members and, if possible, share the model with the relevant interview participant to review any implicit causal links.

### Generalisation of specific issues

When dealing with a large set of transcripts representing the views of diverse stakeholders in a problem situation, there are two key considerations for the modeller: (i) the extent to which highly context-specific issues described by a single individual are relevant to the whole system and (ii) the risk of potentially valuable specific information being watered down or lost as the CLDs are aggregated and simplified. In essence, and in tandem with the use of the-matic coding, the issue relates to the point in the process at which collation and generalisation occurs. Eker and Zimmermann (2016), for example, generalise at the coding stage and use the generalised codes to construct a single consolidated model, while others build multiple models and generalise them in the combination process.

This risk may become apparent when the specificity of a variable is important to the specificity of the intervention. For example, in one transcript, if the variable “Respect for Lived Experience of Inequity and Exclusion as an Expertise” were to be generalised or grouped with other variables as “Respect for Alternative Views,” then the intervention designed may not solve the specific problem originally identified: that particularly hard to reach voices, not just people with alternative views, need to be heard and respected during community engagement. Therefore, if highly context-specific issues are represented as general issues when simplified or combined into aggregated CLDs, it is good practice to capture which interview transcripts the variables were extracted from to ensure traceability and be clear about the steps taken in renaming them. Equally, distinguishing between individual mental models, shared models, and collective models can be useful.

### Language

There is always potential for language to be used in different ways by different interviewees, different ways it might be interpreted and translated into variables and causal connections by the model builder, and different ways the variables might be interpreted by different audiences. There may not be consensus between interviewees on the meaning of words. An example in this research was around the interchangeable use of “participation,” “engagement,” and “consultation” by interviewees discussing public involvement in urban development processes. Some may use them interchangeably; others may see them as distinct activities at different stages of the overall process. There were similar issues with “codesign,” “cocreation,” and “coproduction.” This becomes an issue of generalisation and raises questions as to the most appropriate stage in the process to harmonise, consolidate, and simplify a collective model or the elements from which it is constructed. If independent roles, either the coder or model builder may generalise these terms into a single theme or variable.

Language becomes a key consideration when using relatively simple search queries as a form of semiautomation. In the examples here, the manual variations identified implicit causality that would not have been found by the semiautomated search query. The semiautomated variations also tended to return a high degree of false positives. Words like “so” are used causally and as such cannot be easily ignored from the search function but are most frequently used in a noncausal sense. In the cases used here, the semiautomated variations also returned causal statements and potential variables from subsets of coded data that were not related to the theme of interest. Further work may be required to consider the relationship between the coding process and the model-building stages, particularly when the coding is not conducted exclusively for the purposes of constructing CLDs or similar.

### Efficacy

Taking into account the discussions above, the overarching and most important consideration is the extent to which the method variations help or hinder the study to achieve its ultimate purpose. The use of coded transcripts allows detailed models to be built around thematic areas which can in turn lead to more nuanced policy interventions within the identified themes. This also facilitates the participation of domain experts and thematic stakeholders in extending/validating the resultant structures, although as discussed above, it may be necessary to flex the boundary of the data set extracted through thematic coding to reveal the full context. Acknowledging this limitation, Variations 2 and 4 are most promising for the study in question. Variation 4 seemingly supports some mitigation of biases that emerge from a fully manual process but as applied here, it comes at the cost of overlooking implied causality that do not use specifically causal words. While the use of automated processes may change with the rapid development of language models, machine learning, and artificial intelligence, the evidence reported here suggests the semiautomated variations (Variation 3 and 4) were able to identify more variables and relationships. A more sophisticated process of automatically identifying causal expressions may be required to reduce the number of false positives and increase time efficiency. Again, from the perspective of understanding causal mechanisms sufficiently to design effective policy interventions, the manual variation applied to data sets grouped by thematic coding (Variation 2) appears more suitable.

## Conclusions

Constructing concise yet complete and meaningful causal loop diagrams from a large data set of interview transcripts requires efficient and reliable methods. Such methods often involve the use of thematic coding to segment and extract smaller samples from the overall data set. Others involve elements of automation to seek and identify potential suggestions of causality. Many also utilise elements of consolidating variables and simplifying models at varying stages within the process. These different methods present advantages but also challenges of which the modeller must be aware. Comparing different methods can be difficult due to variation in the description of the steps within these methods across the literature concerning the construction of CLDs from interview transcripts and other text. There is value in developing more consistent language around these processes.

Manually constructing models (i.e. without any automation) from the entire body of text (i.e. with no relation to thematic coding) can be time consuming to the point of being impractical. Condensing the body of text for analysis by selecting a subset of data segments coded with a particular theme/category can save time but may miss important peripheral variables outside the coded data segments. Constructing a model directly from thematic codes, whereby codes directly relate to variable names, can offer significant time savings but requires careful consideration in the coding process and may be inappropriate if the coding was not done with the construction of CLDs in mind. Furthermore, model builders should be aware of cognitive biases when determining implicit causality and employ strategies to address them, particularly in the application of manual approaches to CLD construction.

Decisions regarding the choice of method variation relate to the ultimate purpose of the modelling. The use of coding to identify themes within a large data set can be particularly useful, providing the modellers are also aware of the broader context of the thematically coded data segments within their original transcripts. The relatively simple semiautomated variations considered here do not improve efficiency significantly due to the nuances of natural language producing many false positives (e.g. the use of “so” in a noncausal sense) and utilising implicit causality which the semiautomated function overlooks. In contrast, software commonly used for thematic analysis and model building provide the means to include direct connections between the variables and their origins in the interview transcripts which can overcome the reliance on coding charts that can become laborious and cumbersome for particularly large data sets.

Capturing quantitative characteristics describing the conversion of text to CLDs (e.g. the number of words in the qualitative source data, the number of data segments, the number of variables, the number of microstructures, etc.) was useful for evaluating differences between variations. However, only a small sample was produced in this research. It is therefore recommended that other model builders consider recording and reporting similar quantitative characteristics in the hope that more robust comparisons can be made regarding the construction of CLDs from text. In time this may provide a benchmark with which others can design their own research and assess their models and processes.

## Figures and Tables

**Table 1:** Characteristics of Interviews

Team	Interviews	Interviewees
1 — Local & National Government	24	24
2 — Local Government	16	17
3 — National Government	13	13
4 — Private Sector (Corporate Governance)	13	15
5 — Private Sector (Real Estate)	19	21
6 — Private, Third- and Hybrid-Sector Orgs	22	22
7 — Spatial Planning	16	20
	**123**	**132**

**Table 2:** Characteristics of comparable studies constructing CLDs in public health research

Research paper	No. of interviews	GMB step included?	Characteristics of Final CLD		
			No. of variables	No. of causal links	No. of feedback loops highlighted
Owen *et al.* (2018)	16	No	13	27	6
Baugh Littlejohns et al. (2018)	53	No	19	41	18
Eker et al. (2018)	17	Yes	6	9	2
Clarke et al. (2021)	57	No	21	36	11
Bensberget al. (2021)	31	No	6	17	1

**Table 3:** Example coding chart to inform CLD construction from interview transcripts (coding-chart layout based on Owen *et al*. (2018), populated with data from TRUUD)

Step (1) Coded text data segment			
Text: “By making this better public realm it can help increase office or residential values because it attracts people in.”			
Step (2) Identify “microstructures” of cause variables, effect variables, and their relationships within coded text			
Microstructure	Cause variable	Effect variable	Relationship
Microstructure 1	Public realm improvements	Footfall	Positive
	Footfall	Value of offices or residential buildings	Positive
Step (3) Transform microstructures into words-and-arrow diagrams			
Cause	Effect	Relationship type	Words-and- arrow diagrams
Public realm improvements	Footfall	+ +	Public realm improvements → + Footfall
Footfall	Value of offices or residential buildings	+	Footfall → + Value of offices or residential buildings

**Table 4:** Comparison of processes of causal mapping from text identified in the literature

Description of process	Main tools	Input	Output	Kim and Andersen (2012)	Turner *et al.* (2013b)	Eker and Zimmermann (2016)	Baugh Littlejohns *et al.* (2018)
1. Identifying concepts and discovering themes in the data	Open coding	Raw text data Context for the data	Definition of problem and system boundary Data labelled with themes	Group-level data used Context shared among group members Relevant data segments selected for Step 3	Individual-level data used Context set by researchers Moved to Step 2	Individual-level data used Context set by researchers Coders’ understanding of causal relationships logged to assist Step 3 Moved to Step 2	Individual- and group-level data used Context set by researchers Documents and transcripts coded according to unique coding schema and summary of key findings produced Moved to Step 2
2. Sorting or categorising themes in the data	Axial coding	Data labelled with themes	Data sorted by themes within each stakeholder group	[Despite representation in Kim and Andersen (2012) and Turner *et al.* (2013a), implication is sorting to themes occurs in Step 1 as a second phase of open coding]	Three stakeholder groups identified Moved to Step 3	Four interviewee groups and their domains of action used to define parent nodes of coding tree	
		Data labelled with themes	Coding tree			Coding hierarchy formed according to aggregation of themes into more abstract themes Moved to Step 3	
		Data labelled with themes	Key findings and dominant themes				Key findings found in both documents and transcripts labelled as dominant themes Moved to Step 3
3. Identifying variables and their causal relationships	Open coding Causal links	Data segments	Coding charts	Coding charts from all data segments compiled together Moved to Step 4	Coding charts compiled by themes within in each group Moved to Step 4		
		Coding tree	Coding dictionary			Causal relationships between variables within abstract theme recorded in NVivo Move to Step 5	
		Key findings and dominant themes	[Form of output not reported]				Causal links identified among both dominant themes and key findings Moved to Step 4
4. Transforming text into words-and-arrow diagrams	Causal links Causal maps	Coding charts	Simple words-and-arrow diagrams	One or more words-and-arrow diagrams generated per coding chart Moved to Step 5	Step 4 occurred at the same time as Step 5		One words-and-arrow diagram per interaction
5. Generalising structural representations	Axial coding Causal maps	Simple words-and-arrow diagrams	Final causal map	One causal map generated from the data Moved to Step 6	One causal map generated per stakeholder group Three stakeholder maps synthesised to create one final map Ended here		Feedback loops formed from words-and-arrow diagrams synthesised to create one final map Ended here
		Coding dictionary	Final causal map			One causal map generated per abstract theme Causal maps synthesised to create one final map	
6. Linking maps to the data source	Map/data ID numbers	Coding charts and final causal map Coding dictionary	Data source reference table Data source reference table	Ended here		Relationships between nodes recorded and linked to data sources in NVivo Ended here	

**Table 5:** Comparison of manual and semiautomated CLD construction processes from uncoded and coded transcripts

				Methodological processes			
Description of process	Main tools	Input	Output	Variation 1: Manual construction from uncoded transcripts	Variation 2: manual construction from coded transcripts	Variation 3: semiautomated construction from uncoded transcripts	Variation 4: Semiautomated construction from coded transcripts
1. Discovering themes in the data	Open coding	Raw text data Context for the data	Definition of problem and system boundary Data labelled with themes		Individual-level data used. Context set by researchers. Moved to Step 2		Individual-level data used. Context set by researchers. Moved to Step 2
2. Sorting themes in the data	Axial coding	Data labelled with themes	Coding tree with coded data segments		Themes grouped into higher-level themes forming coding tree, i.e. categories (higher level themes) and subcategories (lower level themes).		Themes grouped into higher-level themes forming coding tree, i.e. categories (higher level themes) and subcategories (lower level themes).
3. Identifying variables and their causal relationships	Open coding Causal links	Raw text data	Microstructure maps	Step 3 occurred at the same time as Step 4			
		Coded data segments Raw text data	Microstructure maps NVivo annotation	[Recording NVivo annotations is possible/optional]	Step 3 occurred at the same time as Step 4	Text search query for words/phrases expressing causal relations. Cause-and-effect variables and direction/polarity of link annotated	
		Coded data segments	NVivo annotation		[Recording NVivo annotations is possible/optional]		Text search query for words/phrases expressing causal relations. Cause-and-effect variables and direction/polarity of link annotated
4. Transforming text into microstructure maps	Causal links Causal maps	Raw text	Microstructure maps	Interview-specific microstructure maps constructed from variables and causal relationships identified in individual transcripts			
		Coded data segments	Microstructure maps		Thematic microstructure maps constructed from variables and causal relationships identified in coded data segments		
		NVivo annotation	Microstructure maps			Microstructure maps of variables and causal links constructed from annotations in individual interview transcripts	Microstructure maps of variables and causal links constructed from annotations in coded segments
5. Generalising structural representations	Axial coding Causal maps	Microstructure maps	Final causal map	Microstructure maps merged to generate one causal map per interview (i.e. an individual mental model) Ended here	Microstructure maps merged to generate one causal map per theme (i.e. a shared mental model) Ended here	Microstructure maps merged to generate one causal map per interview (i.e. an individual mental model)	Microstructure maps merged to generate one causal map per theme (i.e. a shared mental model)
6. Linking maps to the data source	Map and annotation	Final causal map and NVivo annotation	No output	[Link back to NVivo annotation if recorded in Step 3]	[Link back to NVivo annotation if recorded in Step 3]	Link back to NVivo annotation	Link back to NVivo annotation

**Table 6:** Comparison of research design dimensions for alternative coding approaches (adapted from Eker and Zimmermann [2016] based on Turner, Kim, and Andersen, [2013a])

Characteristics	Research design dimension	Kim and Andersen (2012)	Turner *et al.* (2013b)	Eker and Zimmermann (2016)	Baugh Littlejohns *et al.* (2018)	TRUUD approach
Group characteristics	Synchronous vs. asynchronous communication	Synchronous	Asynchronous	Asynchronous	Asynchronous and Synchronous	Asynchronous
	One group vs. many groups	Many groups	Many groups	Many groups	Many groups	Many groups
Data collection characteristics	Context set by researchers vs. by participants	Participants	Researcher	Researcher	Researcher	Researcher
	Data collected by researcher or not	No	Researcher	Researcher	Researcher	Researcher
Coder characteristics	One coder vs. many coders	2^a^	1	1	1	10
	Coder engaged in data collection or not	No	Yes	No	Yes	Yes
Modeller characteristics	Modeller engaged in data collection or not	No	Yes	Yes	Yes	No
	Modeller involved in coding or not	Yes	Yes	Yes	Yes	No

a The number of coders is not specified in the article, so it is assumed that both authors coded the data.

**Table 7:** Summary of CLD uses

Label	CLD use description	Constructed from uncoded transcripts	Constructed from coded transcripts
U1	Examples for group model building workshops	✓	
U2	Reflections on individual interviewee mental models	✓	
U3	Basis for thematic workshops discussion		✓
U4	Identification of potential intervention areas		✓
U5	Identification of gaps in data for additional interviews		✓

**Table 8:** Quantitative characteristics of variations on the method applied to construct CLDs

CLD construction approach	Method variation	No. of words in source data^a^	No. of variables	No. of microstructures	No. of feedback loops
Manual	1	7066	123	27	6
Manual	1	10,552	98	15	0
Manual	2	8632	133	26	8
Semiautomated	3	5680	59	16	0
Semiautomated	3	10,071	60	20	1
Semiautomated	3	6171	55	18	0

a The number of words includes both interviewer and interviewee dialogue plus indicator of who is speaking.
